# Supplementary material for: Assessment of plum rain’s impact on power system emissions in Yangtze-Huaihe River basin of China
Source: Nat Commun. 2021 Oct 25;12:6156. doi: 10.1038/s41467-021-26358-w (PMC8545941; doi:10.1038/s41467-021-26358-w)
Supplement: Supplementary file 1 — Supplementary Information [file 41467_2021_26358_MOESM1_ESM.pdf]

Supplementary Information

Assessment of plum rain's impact on power system emissions in Yangtze-Huaihe River basin of China

Guangsheng Pan, Qinran Hu, Wei, Gu, Shixing Ding, Haifeng Qiu, Yuping Lu

Supplementary Figures

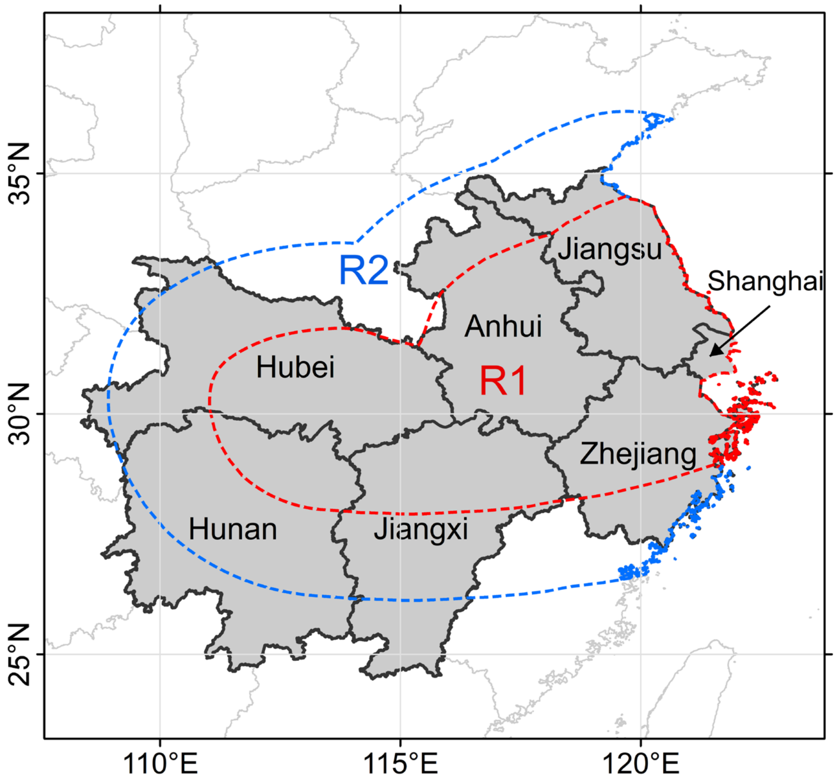

Supplementary Figure 1 Boundaries of the affected region (R1) and the unaffected surrounding region (R2). The region enclosed within the red dashed line is R1. The region between the red dashed line and the blue dashed line is R2.

a

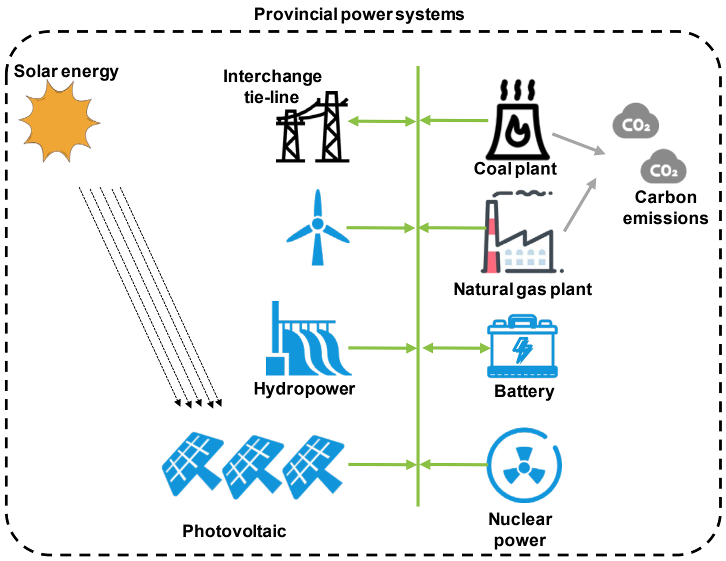

b

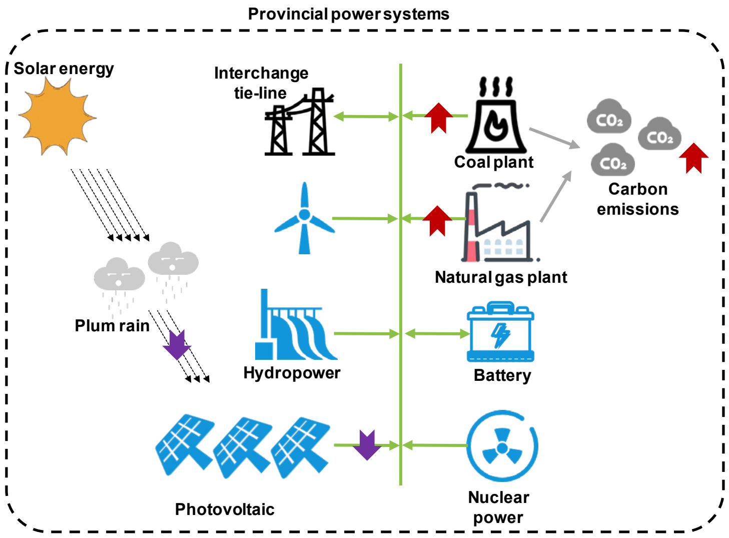

Supplementary Figure 2 Schematic diagram of plum rain's impact on provincial power systems. a Operation of provincial power systems before plum rain. **b** Operation of provincial power systems during plum rain period. The blue and red arrows represent falling and rising, respectively. The black dotted line is the solar irradiance. The solid green line is power flow, and the solid gray line is carbon emissions.

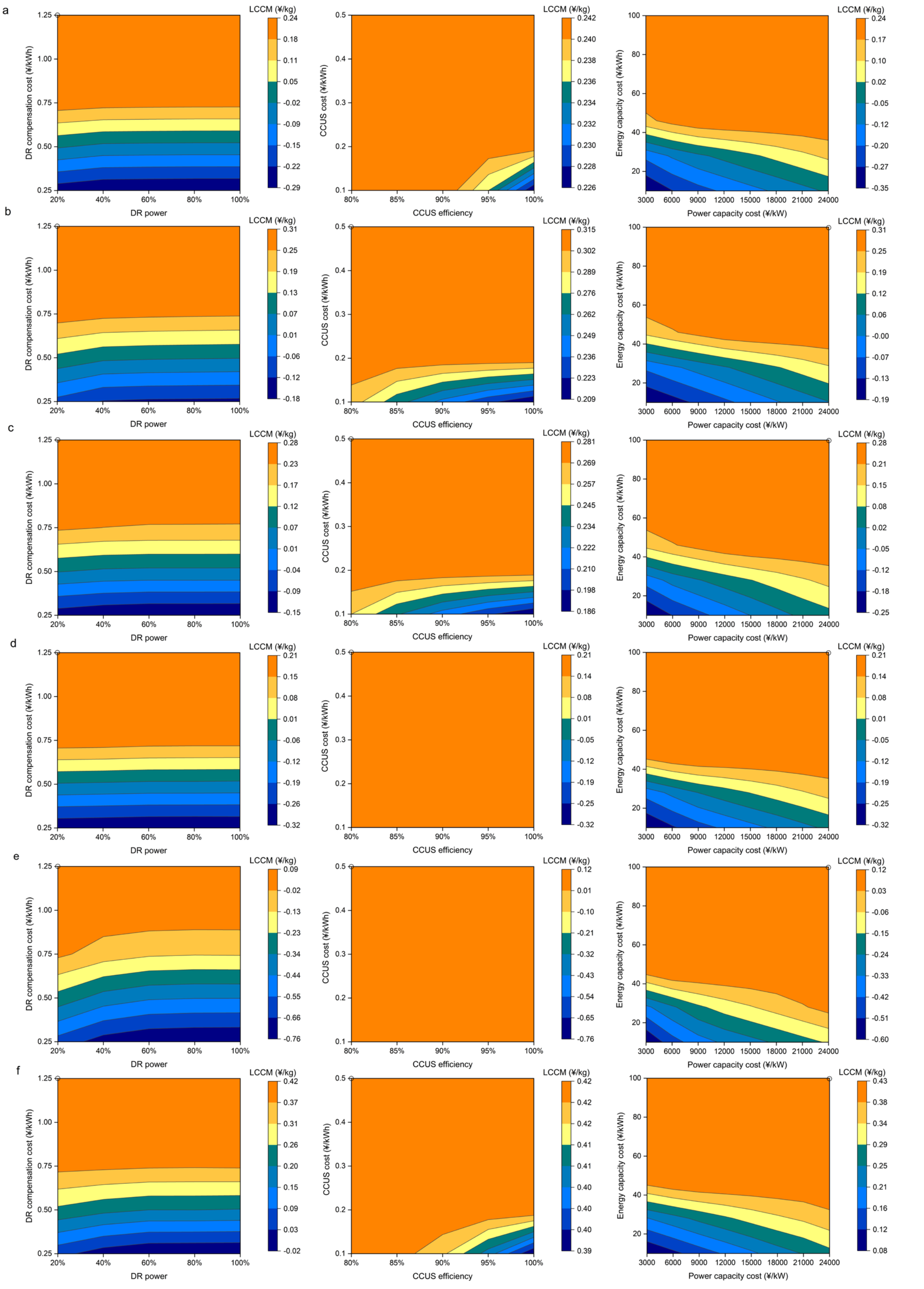

Supplementary Figure 3 Levelized cost of CO_2 mitigation (LCCM) of coal power to natural gas power (C2N)+demand response (DR), C2N+carbon capture, utilization and storage (CCUS), and C2N+long-duration (LD) under different techno-economic parameters. **a** Jiangsu. **b** Anhui. **c** Jiangxi. **d** Hubei. **e** Shanghai. **f** Hunan. In each province, LCCM of C2N+DR, C2N+CCUS, and C2N+LD are given from left to right.

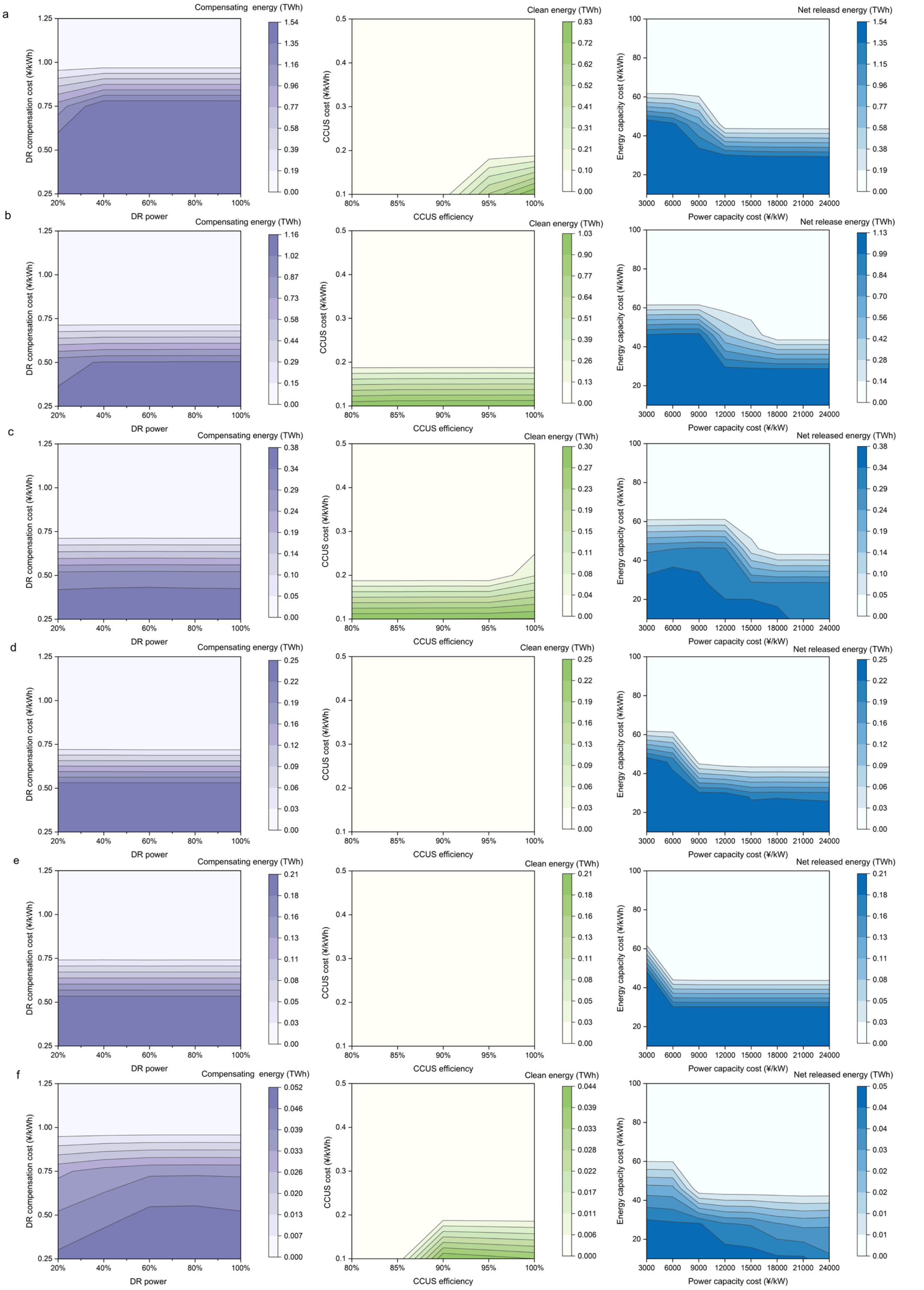

Supplementary Figure 4 Compensating energy of coal power to natural gas power (C2N)+demand response (DR), C2N+carbon capture, utilization and storage (CCUS), and C2N+long-duration (LD) under different techno-economic parameters. **a** Jiangsu. **b** Anhui. **c** Jiangxi. **d** Hubei. **e** Shanghai. f Hunan. In each

province, compensating energy of C2N+DR, C2N+CCUS, and C2N+LD are given from left to right.

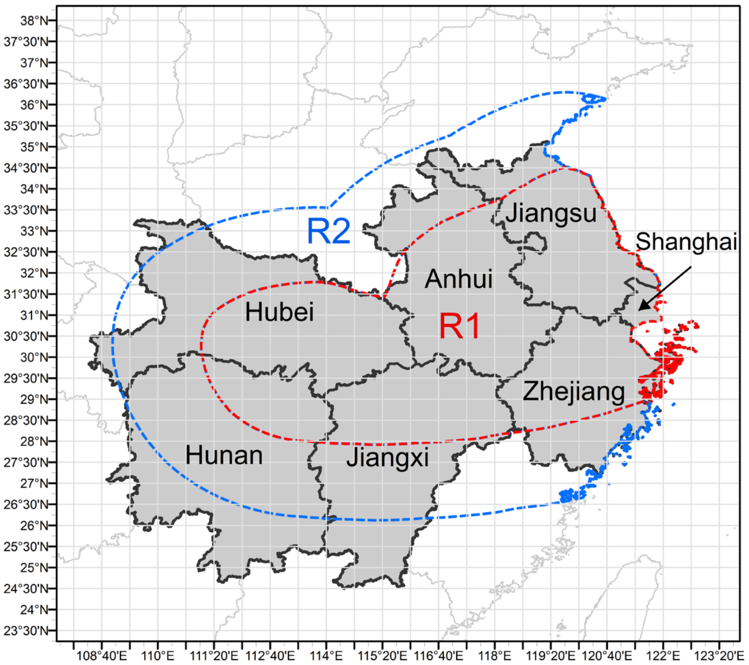

Supplementary Figure 5 Spatial resolution of 1/2° latitude by 2/3° longitude to calculate photovoltaic capacity factor. R1 denotes the affected region and R2 denotes the unaffected surrounding region.

Supplementary Tables

Supplementary Table 1. Predicted change trends of different generators demand.[1]

Type	2020	2030	2040	2050
Coal power	1	1.3	1.09	0.74
Natural gas power	1	1.9	3	3.9
Photovoltaic	1	2.87	4.94	6.56
Interchange tie-line	1	1.4	1.8	2.2
Power load	1	1.49	1.74	1.83

Supplementary Table 2. Area ratio, impact degree, and photovoltaic (PV) capacity factor (CF) in each affected province/municipality

Province	Area ratio	θ_P^{MEAN}	PV capacity factor[2]
Jiangsu	80%	7.2 %	0.17
Anhui	77%	7.0 %	0.17
Zhejiang	70%	10.2%	0.156
Jiangxi	45%	7.8 %	0.156
Hubei	53%	4.3%	0.172
Shanghai	100%	10.2%	0.164
Hunan	18%	5.0 %	0.162

Supplementary Table 3. Economic and technical parameters of clustered power plant types.[3]

Plant type	Capacity (MW)	Startup/shutdown time (h)
Coal generator	≤1000	16
	600≤ and < 1000	8
	300≤ and < 600	7
	200≤ and < 300	6
	100≤ and < 200	5
	6≤ and < 100	2
Natural gas generator	300≤ and < 600	7
	200≤ and < 300	6
	100≤ and < 200	5
	6≤ and < 100	2

* The startup and shutdown cost of generating unit is assumed to be proportional to the capacity.[4] For example, a 500-MW unit has a startup and shutdown cost of 500000 ¥.

Supplementary Table 4. Fuel cost and emission factor by power plant type.

Plant type	Fuel cost (¥/kWh)[5]	Emission factor (kg/kWh)[6]
Coal	0.37	1.22945
Natural gas	0.65	0.3756

Supplementary Note 1. Detailed description of Global Solar Energy Estimator (GSEE) model[2]

According to the study by Pfenninger et al[2], the power output of PV modules depends on the in-plane irradiance G and module temperature T_{mod}:

$$P^{\text{PV}}\left(G,T_{\text{mod}}\right)=P_{\text{STC}}^{\text{PV}}\cdot\frac{G}{G_{\text{STC}}}\cdot\eta_{\text{rel}}\left(G',T'\right) \tag{1a}$$

where $P_{\text{STC}}^{\text{PV}}$ is the power output at standard test conditions (STC) with in-plane irradiance G_{STC} of 1000W/m^2 and module temperature $T_{\text{mod\_STC}}$ of 25 °C. The hourly instantaneous relative efficiency η_{rel}, depending on the instantaneous irradiance and temperature, is given by[7]

$$\eta_{\text{rel}}\left(G',T'\right)=1+k_1\ln G'+k_2\left(\ln G'\right)^2+T'\left[k_3+k_4\ln G'+k_5\left(\ln G'\right)^2\right]+k_6T'^2 \tag{1b}$$

where $G'=\frac{G}{G_{\text{STC}}}$ and $T'=T_{\text{mod}}-T_{\text{mod\_STC}}$ are normalized parameters to STC values. k_1-k_6 are coefficients determined by experimental data. T_{mod} can be further calculated by the ambient temperature and irradiation G as follows:

$$T_{\text{mod}}=T_{\text{amb}}+c_TG \tag{1c}$$

where c_T represents how much PV module is heated by irradiation G.

The in-plane irradiance G includes the direct and diffuse plane irradiance (G_{dir} and G_{dif}), which can be computed from the global direct and diffuse irrad iance (I_{dir} and I_{dif}) by

$$G_{dir}=\frac{I_{dir}\cdot\cos(\alpha)}{\cos(\frac{\pi}{2}-a_s)} \tag{1d}$$

$$G_{dif}=I_{dif}\cdot\frac{1+\cos(\Sigma)}{2}+a\cdot\left(I_{dir}+I_{dif}\right)\cdot\frac{1-\cos(\Sigma)}{2} \tag{1f}$$

where α is the plane incidence angle, calculated by (1g) with a fixed tilt angle. a_{s} is the sun azimuth angle, a is the surface albedo (a=0.3).

$$\alpha=\arccos\left(\sin(h)\cdot\cos(\Sigma)+\cos(h)\cdot\sin(\Sigma)+\cos(a_p-a_s)\right) \tag{1g}$$

where h is sun altitude, a_p is panel azimuth, and a_s is sun azimuth angle.

Σ is the plane tilt in degrees, calculated by

$$\Sigma=1.3793+lat\cdot\left(1.2011+lat\cdot(-0.014404+lat\cdot0.000080509)\right) \tag{1h}$$

where lat is the latitude in degrees. Considering that the plum rain-affected areas occur near latitude 30°N, we set the optimum tilt angle of the fixed-tilt system to 26.6 degrees, following the study by Chen et al.[8] In the end, the system loss is set to the default value of 0.1.

Supplementary Note 2. Comparison of various pathways.

Based on the above optimization results, we further compare four pathways in offsetting the incremental CO_2 emissions caused by plum rain. First, we use the C^{ES}, which is obtained in the first optimization, as a known quantity in the following optimization model to eliminate the influence of electric storage. Then, we also add the calculated ΔE and PV reduction ΔP^{PV} caused by plum rain in the following optimization model to measure the capacity required for each pathway.

The PV reduction ΔP^{PV} can be calculated as

$$\Delta P^{PV} = \sum_{l_1 \in \Phi_{R1}^{PV}} \sum_{t \in \Phi^{T}} P_{l_1}^{PV}(t)^{(2)} - \sum_{l_1 \in \Phi_{R1}^{PV}} \sum_{t \in \Phi^{T}} P_{l_1}^{PV}(t)^{(1)} \tag{2}$$

where $P_{l_1}^{PV}(t)^{(1)}$ and $P_{l_1}^{PV}(t)^{(2)}$ are the actual output power of l_1-*th* PV generator in the affected region at the first and second optimization, respectively.

1) Coal power to natural gas power (C2N)

The objective function is revised as follows:

$$\begin{aligned}\min_{\mathbf{X}'} C = &\sum_{i \in \Phi^{TG}} \sum_{t \in \Phi^{T}} \left[c_i^{TG} P_i^{TG}(t) + c_i^{U} Y_i(t) + c_i^{D} Z_i(t) \right] \\ &+ \varphi^{PV} \sum_{l_1 \in \Phi_{R1}^{PV}} \sum_{t \in \Phi^{T}} \left[\rho_{R1}^{PV}(t) C_{l_1}^{PV} - P_{l_1}^{PV}(t) \right] \\ &+ \varphi^{PV} \sum_{l_2 \in \Phi_{R2}^{PV}} \sum_{t \in \Phi^{T}} \left[\rho_{R2}^{PV}(t) C_{l_2}^{PV} - P_{l_2}^{PV}(t) \right] \\ &+ c_{PR}^{ES} C^{ES} + \varphi^{Loss} \sum_{t \in \Phi^{T}} P^{Loss}(t) \end{aligned} \tag{3a}$$

where $\mathbf{X}'$ is the decision variable set $\mathbf{X}$ that does not contain C^{ES}, C is the total cost expressed in formula (2) in the main manuscript, except that C^{ES} is a known quantity.

In the original clustered unit commitment (CUC) model (2)—(17) of the main manuscript, the following carbon emission constraints are added to offset the incremental CO_2 emissions by converting coal power to gas power.

$$e^{C} \sum_{p \in \Phi_{C}^{TG}} \sum_{t \in \Phi^{T}} P_p^{TG}(t) + e^{N} \sum_{q \in \Phi_{N}^{TG}} \sum_{t \in \Phi^{T}} P_q^{TG}(t) = E^{(2)} \tag{3b}$$

where $E^{(2)}$ is the CO_2 emissions in the second optimization removing the negative effects of plum rain.

2) Demand response (DR) program

First, adding the total DR cost in the objective function:

$$\min_{\mathbf{X}'} C + c^{DR} \sum_{t \in \Phi^{T}} P^{DR}(t) \tag{4a}$$

where $P^{DR}(t)$ is the load curtailment at time t, and c^{DR} is the unit compensation cost for DR program.

Then, using constraint (4b) to replace constraint (3) in the main manuscript, and adding the following constraints (4c-4e) in the CUC model.

$$\begin{aligned}&\sum_{i\in\Phi^{\mathrm{TG}}}P_i^{\mathrm{TG}}(t)+\sum_{k\in\Phi^{\mathrm{WT}}}P_k^{\mathrm{WT}}(t)+\sum_{l_1\in\Phi_{\mathrm{R1}}^{\mathrm{PV}}}P_{l_1}^{\mathrm{PV}}(t)+\sum_{l_2\in\Phi_{\mathrm{R2}}^{\mathrm{PV}}}P_{l_2}^{\mathrm{PV}}(t)+\sum_{m\in\Phi^{\mathrm{RG}}}P_m^{\mathrm{RG}}(t)\\&+\sum_{n\in\Phi^{\mathrm{TL}}}P_n^{\mathrm{TL}}(t)+P^{\mathrm{ES-}}(t)-P^{\mathrm{ES+}}(t)+P^{\mathrm{DR}}(t)=P^{\mathrm{Load}}(t)-P^{\mathrm{Loss}}(t),\ \forall t\in\Phi^{\mathrm{T}}\end{aligned}\tag{4b}$$

$$0\le P^{\mathrm{DR}}(t)\le\beta\max\left(P^{\mathrm{Load}}(t)\right),\ \forall t\in\Phi^{\mathrm{T}}\tag{4c}$$

$$\sum_{t\in\Phi^{\mathrm{T}}}P^{\mathrm{DR}}(t)\le\Delta P^{\mathrm{PV}}\tag{4d}$$

$$e^{\mathrm{C}}\sum_{p\in\Phi_{\mathrm{C}}^{\mathrm{TG}}}\sum_{t\in\Phi^{\mathrm{T}}}P_p^{\mathrm{TG}}(t)+e^{\mathrm{N}}\sum_{q\in\Phi_{\mathrm{N}}^{\mathrm{TG}}}\sum_{t\in\Phi^{\mathrm{T}}}P_q^{\mathrm{TG}}(t)=E^{(2)}\tag{4e}$$

Equation (4b) defines the supply and demand power balance considering DR program. Constraint (4c) bounds the minimum and maximum outputs of DR program, where β denotes the proportion of load curtailment to the total electric load. Constraint (4d) imposes the total load curtailment during the plum rain period less than the PV reductions. In the end, CO_2 emission constraint (4e) is added to offset the incremental CO_2 emissions.

3) Carbon capture, utilization and storage (CCUS)

Similarly like DR program, the objective function is changed as

$$\min_{\mathbf{X}'}C+c^{\mathrm{CCUS}}P^{\mathrm{CCUS}}\tag{5a}$$

where P^{CCUS} is the power capacity of coal generators installed with CCUS, and c^{CCUS} is the capture cost per one unit of electricity.

Then, using constraint (5b) and (5c) to replace constraints (3) and (4) in the main manuscript, and adding the following constraints (5d)-(5f) in the clustered unit commitment model.

$$\begin{aligned}&\sum_{i\in\Phi^{\mathrm{TG}}}P_i^{\mathrm{TG}}(t)+\sum_{k\in\Phi^{\mathrm{WT}}}P_k^{\mathrm{WT}}(t)+\sum_{l_1\in\Phi_{\mathrm{R1}}^{\mathrm{PV}}}P_{l_1}^{\mathrm{PV}}(t)+\sum_{l_2\in\Phi_{\mathrm{R2}}^{\mathrm{PV}}}P_{l_2}^{\mathrm{PV}}(t)+\sum_{m\in\Phi^{\mathrm{RG}}}P_m^{\mathrm{RG}}(t)\\&+\sum_{n\in\Phi^{\mathrm{TL}}}P_n^{\mathrm{TL}}(t)+P^{\mathrm{ES-}}(t)-P^{\mathrm{ES+}}(t)-\sigma^{\mathrm{CCUS}}P^{\mathrm{CCUS}}=P^{\mathrm{Load}}(t)-P^{\mathrm{Loss}}(t),\ \forall t\in\Phi^{\mathrm{T}}\end{aligned}\tag{5b}$$

$$\begin{aligned}&\sum_{i\in\Phi^{\mathrm{TG}}}P_{i,\max}^{\mathrm{TG}}U_i(t)+\sum_{m\in\Phi^{\mathrm{RG}}}P_m^{\mathrm{RG}}(t)+\sum_{n\in\Phi^{\mathrm{TL}}}P_n^{\mathrm{TL}}(t)+S^{\mathrm{ES}}(t-1)\\&-\sigma^{\mathrm{CCUS}}P^{\mathrm{CCUS}}\ge P^{\mathrm{Load}}(t)+RS(t),\ \forall t\in\Phi^{\mathrm{T}}\end{aligned}\tag{5c}$$

$$\left(\eta^{\mathrm{CCUS}}-\sigma^{\mathrm{CCUS}}\right)P^{\mathrm{CCUS}}\le\Delta P^{\mathrm{PV}}\tag{5d}$$

$$e^{\mathrm{C}}\sum_{p\in\Phi_{\mathrm{C}}^{\mathrm{TG}}}\sum_{t\in\Phi^{\mathrm{T}}}P_p^{\mathrm{TG}}(t)-\eta^{\mathrm{CCUS}}e^{\mathrm{C}}P^{\mathrm{CCUS}}T+e^{\mathrm{N}}\sum_{q\in\Phi_{\mathrm{N}}^{\mathrm{TG}}}\sum_{t\in\Phi^{\mathrm{T}}}P_q^{\mathrm{TG}}(t)=E^{(2)}\tag{5e}$$

Equations (5b) and (5c) show the power balance and system reserve requirements considering CG with CCUS, respectively, where η^{CCUS} is the CCUS equipment efficiency, σ^{CCUS} is the ratio of consumed power by CCUS equipment. Constraint (5d) imposes the clean energy produced by plum rain period less than the PV reductions. In the end, CO_2 emission constraint (5e) is added to offset the incremental CO_2 emissions.

4) Long-duration (LD) energy storage

Similarly, the objective function of the UC model considering long-duration storage integration is given as follows:

$$\min_{\mathbf{X}'} C+\frac{T}{8760}\frac{r\cdot(1+r)^{n^{\mathrm{LD}}}}{(1+r)^{n^{\mathrm{LD}}}-1}(c_{\mathrm{P}}^{\mathrm{LD}}P_{\max}^{\mathrm{LD}}+c_{\mathrm{E}}^{\mathrm{LD}}S_{\max}^{\mathrm{LD}}) \tag{6a}$$

where $P_{\max}^{\mathrm{LD}}$ and $S_{\max}^{\mathrm{LD}}$ are the installed power capacity and energy capacity of LD, respectively. $c_{\mathrm{P}}^{\mathrm{LD}}$ and $c_{\mathrm{E}}^{\mathrm{LD}}$ are the power capacity cost and energy capacity cost of LD, respectively. r and n^{LD} are the discount rate and lifetime of LD, respectively.

Then, using constraint (6b) to replace constraint (3) in the main manuscript, and adding the following constraints (6c)-(6g) in the CUC model.

$$\begin{aligned}&\sum_{i\in\Phi^{\mathrm{TG}}}P_i^{\mathrm{TG}}(t)+\sum_{k\in\Phi^{\mathrm{WT}}}P_k^{\mathrm{WT}}(t)+\sum_{l_1\in\Phi_{\mathrm{R1}}^{\mathrm{PV}}}P_{l_1}^{\mathrm{PV}}(t)+\sum_{l_2\in\Phi_{\mathrm{R2}}^{\mathrm{PV}}}P_{l_2}^{\mathrm{PV}}(t)+\sum_{m\in\Phi^{\mathrm{RG}}}P_m^{\mathrm{RG}}(t)\\&+\sum_{n\in\Phi^{\mathrm{TL}}}P_n^{\mathrm{TL}}(t)+P^{\mathrm{ES-}}(t)-P^{\mathrm{ES+}}(t)+P^{\mathrm{LD-}}(t)-P^{\mathrm{LD+}}(t)=P^{\mathrm{Load}}(t)-P^{\mathrm{Loss}}(t),\forall t\in\Phi^{\mathrm{T}}\end{aligned} \tag{6b}$$

$$0\le P^{\mathrm{LD+}}(t)\le P_{\max}^{\mathrm{LD}},\forall t\in\Phi^{T} \tag{6c}$$

$$0\le P^{\mathrm{LD-}}(t)\le P_{\max}^{\mathrm{LD}},\forall t\in\Phi^{T} \tag{6d}$$

$$S^{\mathrm{LD}}(t)=S^{\mathrm{LD}}(t-1)+\eta^{\mathrm{LD+}}P^{\mathrm{LD+}}(t)-P^{\mathrm{LD-}}(t)/\eta^{\mathrm{LD-}},\forall t\in\Phi^{T} \tag{6e}$$

$$0\le S^{\mathrm{LD}}(t)\le S_{\max}^{\mathrm{LD}},\forall t\in\Phi^{T} \tag{6f}$$

$$S^{\mathrm{LD}}(0)=S_{\max}^{\mathrm{LD}} \tag{6g}$$

$$\sum_{t\in\Phi^{\mathrm{T}}}P^{\mathrm{LD-}}(t)-\sum_{t\in\Phi^{\mathrm{T}}}P^{\mathrm{LD+}}(t)\le\Delta P^{\mathrm{PV}} \tag{6h}$$

$$e^{\mathrm{C}}\sum_{p\in\Phi_{\mathrm{C}}^{\mathrm{TG}}}\sum_{t\in\Phi^{\mathrm{T}}}P_p^{\mathrm{TG}}(t)+e^{\mathrm{N}}\sum_{q\in\Phi_{\mathrm{N}}^{\mathrm{TG}}}\sum_{t\in\Phi^{\mathrm{T}}}P_q^{\mathrm{TG}}(t)=E^{(2)} \tag{6i}$$

Equation (6b) defines the supply and demand power balance considering LD storage. Constraints (6c)-(6f) impose power capacity and energy constraints, respectively, on the charging, discharging, and storage levels of LD storage, where $P^{\mathrm{LD+/-}}(t)$ is the hourly charging/discharging power of LD storage, $S^{\mathrm{LD}}(t)$ is the hourly storage levels of LD storage, and $\eta^{\mathrm{LD+/-}}$ is the charging/discharging efficiency of LD storage. Constraints (6g) guarantees that the LD stores enough energy before the rainy season. Constraint (6h) imposes the net released energy during the plum rain period less than the PV reductions. In the end, CO_2 emission constraint (6i) is added to offset the incremental CO_2 emissions.

References

1. State Grid Energy Research Institute. *China Energy & Electricity Outlook* (China Electric Power Press, 2019).
2. Pfenninger, S. & Staffell, I. Long-term patterns of European PV output using 30 years of validated hourly reanalysis and satellite data. *Energy* **114**, 1251–1265 (2016).
3. China Electricity Council. *China Electric Power statistical yearbook 2020.* http://www.stats.gov.cn/tjsj/tjcbw/202103/t20210329_1815748.html (2021).
4. Zhong, H., Xia, Q., Chen, Y., Kang, C. Energy-saving generation dispatch toward a sustainable electric power industry in China. *Energy policy* **83**, 14-25 (2015).
5. Chen, X., McElroy M. B., and Kang, C. Integrated Energy Systems for Higher Wind Penetration in China: Formulation, Implementation, and Impacts. *IEEE Trans. on Power Syst.* **33**, 1309-1319 (2018).
6. Chen, X., Zhang, H., Xu, Z. et al. Impacts of fleet types and charging modes for electric vehicles on emissions under different penetrations of wind power. *Nat Energy* **3**, 413–421 (2018).
7. Huld, T., Gottschalg, R., Beyer H. G., Topič, M. Mapping the performance of PV modules, effects of module type and data averaging. *Sol. Energy* **84(2)**, 324-338 (2010).
8. Chen, S., Lu, X., Miao Y., et al. The potential of photovoltaics to power the belt and road initiative. *Joule* **3**, 1895–1912 (2019).
